# Tobacco smoking and depressive symptoms in Chinese middle-aged and older adults: Handling missing values in panel data with multiple imputation

**DOI:** 10.3389/fpubh.2022.913636

**Published:** 2022-08-26

**Authors:** Xiahua Du, Rina Wu, Lili Kang, Longlong Zhao, Changle Li

**Affiliations:** ^1^College of Humanities Education, Inner Mongolia Medical University, Hohhot, China; ^2^The Affiliated Hospital of Inner Mongolia Medical University, Hohhot, China; ^3^School of Health Management, Inner Mongolia Medical University, Hohhot, China

**Keywords:** smoking, depressive symptoms, multiple imputation, China, missing value

## Abstract

**Introduction:**

The high co-occurrence of tobacco smoking and depression is a major public health concern during the novel coronavirus disease-2019 pandemic. However, no studies have dealt with missing values when assessing depression. Therefore, the present study aimed to examine the effect of tobacco smoking on depressive symptoms using a multiple imputation technique.

**Methods:**

This research was a longitudinal study using data from four waves of the China Health and Retirement Longitudinal Study conducted between 2011 and 2018, and the final sample consisted of 74,381 observations across all four waves of data collection. The present study employed a multiple imputation technique to deal with missing values, and a fixed effects logistic regression model was used for the analysis.

**Results:**

The results of fixed effects logistic regression showed that heavy smokers had 20% higher odds of suffering from depressive symptoms than those who never smoked. Compared to those who never smoked, for short-term and moderate-term quitters, the odds of suffering from depressive symptoms increased by 30% and 22%, respectively. The magnitudes of the odds ratios for of the variables short-term quitters, moderate-term quitters, and long-term quitters decreased in absolute terms with increasing time-gaps since quitting. The sub-group analysis for men and women found that heavy male smokers, short-term and moderate-term male quitters had higher odds of suffering from depressive symptoms than those who never smoked. However, associations between smoking status and depressive symptoms were not significant for women.

**Conclusions:**

The empirical findings suggested that among Chinese middle-aged and older adults, heavy smokers and short-term and moderate-term quitters have increased odds of suffering from depressive symptoms than those who never smoked. Moreover, former smokers reported that the probability of having depressive symptoms decreased with a longer duration since quitting. Nevertheless, the association between depressive symptoms and smoking among Chinese middle-aged and older adults is not straightforward and may vary according to gender. These results may have important implications that support the government in allocating more resources to smoking cessation programs to help middle-aged and older smokers, particularly in men.

## Introduction

Tobacco smoking is one of the biggest public health threats, resulting in more than 7 million deaths a year worldwide (1). In China, 50.5% of adult men were current smokers in 2018, although the figure was only 2.1% for adult women. The prevalence of current smoking was 30.2% among adults aged 45–64 years and 23.1% among adults aged 65 years or older, implying that there are more than 163 million middle-aged and older adults who smoke in China. Even though tobacco smoking has been proven to be a major cause of diseases such as cancers, heart diseases, and respiratory diseases, only 16.1% of current smokers in China plan to quit smoking within 12 months (2). Meanwhile, depression is currently becoming a significant public health problem, with more than 300 million people estimated to suffer from depression worldwide (3). The prevalence of depressive symptoms among older adults was 20.0%. Depression in late life is associated with an elevated risk of morbidity and suicide and decreased physical and cognitive functioning (4, 5).

The novel coronavirus disease 2019 pandemic has led to adverse changes in health behaviors such as smoking and physical activity and widespread mental disorders such as depression and anxiety (6, 7). The high co-occurrence of tobacco smoking and depression is a major public health concern during this unprecedented crisis. The reciprocal relationships between tobacco smoking and depression have been widely documented; for example, depression is associated with subsequent smoking behavior, and smoking exposure is associated with subsequent depression (8–12). Several previous studies have demonstrated an association between tobacco smoking and depression: tobacco smoking increases the risk of depressive symptoms. Such an association has been shown across different age groups, such as adolescents (13), adults (14, 15), middle-aged and older adults (16, 17), and elderly people (18). Conversely, few studies have shown that tobacco smoking reduces depressive symptoms (19, 20). Furthermore, other studies have shown that depression predicts the persistence of tobacco smoking (21–25).

Previous studies document that the association between depressive symptoms and smoking is relatively stable across ages (26). However, the prevalence of both depression and smoking peaks in adolescence (27). Most studies on depression and smoking were conducted during adolescence. Few studies have explicitly focused on middle-aged and older adults (20, 28). Across the lifespan, a more modest peak in the prevalence of depression occurs in the fifth and sixth decades (29). Moreover, middle-aged and older smokers are an underserved population for smoking cessation interventions (28). Therefore, this study analyzes the association between depressive symptoms and smoking, focusing on middle-aged and older adults.

Past studies provide evidence of the association between depressive symptoms and smoking among Chinese middle-aged and older adults. On the one hand, smoking had higher odds of suffering from depressive symptoms (30); women smokers showed a higher likelihood of suffering from depressive symptoms (31). On the other hand, smokers were less likely to develop depressive symptoms (20); formerly smoking behavior was inversely associated with the risk of depressive symptoms (32). The results show a negative association between depressive symptoms and smoking using the data from the China Health and Retirement Longitudinal Study (CHARLS). In the CHARLS, there were 1,965 missing 10-item Center for Epidemiologic Studies Depression Scale (CESD-10) baseline scores and 2,888 missing CESD-10 follow-up scores among respondents in both interviews. Therefore, the CESD-10 items with missing values may lead to conflicting findings. As we know, missing values are a common challenge in most social research studies, and the problem is often pronounced in studies using self-rated instruments. Similarly, previous studies on depression may have encountered the problem of missing values (33, 34). Missing values reduce statistical power, cause bias in the estimation of the parameters, and lessen the representativeness of the samples (35).

Missingness mechanisms were first introduced by Rubin (36). Rubin distinguished three fundamental missing-values mechanisms: missing completely at random (MCAR), missing at random (MAR), and missing not at random (MNAR). MCAR occurs when the missingness is unrelated to the observed and unobserved value for a unit. Under an MAR mechanism, the probability of a missing value for an item may depend on observed data but not on unobserved data. MNAR means that the probability of missingness depends on the underlying value of an item. Many researchers adopt a listwise deletion approach (complete-case analysis) to deal with missing values. This approach is based on the assumption of MCAR. However, this assumption is sometimes difficult to justify in practice (37). Therefore, various imputation methods have been developed to compensate for missing values in survey data. The methods include random selection, preceding question, question mean, individual mean, single regression, and multiple imputation. Multiple imputation is the most accurate technique for dealing with missing values when assessing depressive symptoms (33).

Overall, the difficulty of estimating the effect of smoking status on depression has led to conflicting findings in the general population. Moreover, although the association between tobacco smoking and depression has been documented, thus far, there have been no studies dealing with missing values when assessing depression. To bridge these gaps, the present study is aimed to examine the effect of tobacco smoking on depressive symptoms using panel data with a multiple imputation technique.

## Methods

### Data source

The data used in this study were obtained from the China Health and Retirement Longitudinal Study (CHARLS), launched by the National School of Development of Peking University. The CHARLS sample was drawn from 28 provinces, 150 counties/districts, and 450 villages/residential committees. Multistage stratified sampling with a probability proportional to size was used for the survey. More details on the sampling procedure and data collection process are available in the study by Zhao et al. (38). The CHARLS questionnaires include sections on household information, demographic background, health status and physical functioning, health care and insurance, work, retirement, pension, income, expenditure, assets, housing characteristics, etc.

The CHARLS is a biennially longitudinal survey of Chinese families and individuals aged older than 45 years. In the first wave in 2011, 17,337 persons (older than 45 years old) were successfully interviewed; in the three waves of full sample follow-up surveys in 2013, 2015, and 2018, 18,248 persons, 20,083 persons, and 19,584 persons were successfully interviewed, respectively. The measured variables and their respective percentages of missing values are presented in Table 1. After eliminating the measured variables with missing values <1% (871 persons), the final sample consisted of a total of 74,381 persons for the data collection waves, 58,854 with no missing values and an additional 15,527 with missing values in at least one of the measured variables.

**Table 1:** Frequencies and percentages of missing values for the measured variables.

**Variables**	**Complete response, *N***	**Complete %**	**Missing response, *N***	**Missing %**
**Depressive symptoms**
CESD-10-01	67,454	89.64	7,798	10.36
CESD-10-02	66,661	88.58	8,591	11.42
CESD-10-03	67,362	89.52	7,890	10.48
CESD-10-04	67,413	89.58	7,839	10.42
CESD-10-05	65,694	87.30	9,558	12.70
CESD-10-06	68,008	90.37	7,244	9.63
CESD-10-07	68,081	90.47	7,171	9.53
CESD-10-08	67,726	90.00	7,526	10.00
CESD-10-09	67,704	89.97	7,548	10.03
CESD-10-10	67,376	89.53	7,876	10.47
Smoking status	66,228	88.01	9,024	11.99
Sex	75,203	99.93	49	0.07
Age	74,830	99.44	422	0.56
Educational attainment	73,461	97.62	1,791	2.38
Marital status	75,145	99.86	107	0.14
Rural residency	72,725	96.64	2,527	3.36
Self-rated health	72,089	95.80	3,163	4.20
Functional limitations	74,781	99.37	471	0.63
Chronic conditions	72,614	96.49	2,638	3.51
Drinking	74,607	99.14	645	0.86

“Don't know,” “Refused,” and “Blank” equal missing.

### Measurements

#### Depressive symptoms

The CESD-10 was used to assess depressive symptoms in the CHARLS questionnaire. First, the CESD-10 was developed from the full-length version of the 20-item Center for Epidemiologic Studies Depression Scale (CESD-D), which was designed as a screening instrument for depressive symptoms in older adults (39). Second, the CESD-10 indicated good predictive accuracy when compared to the CESD-D. Last, the CESD-10 has shown reasonable validity and reliability in the Chinese population and adequate validity across a range of ages in longitudinal studies (40, 41).

In the CHARLS, each respondent was asked, “How often have you felt or behaved this way during the last week?”. The survey consists of 10 items (e.g., I felt depressed, I felt fearful, my sleep was restless, and I was happy), which can be rated on a 4-point Likert scale from 0 (<1 day) to 3 (5–7 days). The range of the CESD-10 total score is 0–30, and a cutoff score of 10 or higher indicates the presence of significant depressive symptoms (39). Therefore, the variable of depressive symptoms was set as a dichotomous variable that equaled 1 if the individual self-rated CESD-10 score was equal to or >10 and equaled 0 if otherwise.

#### Smoking status

In the CHARLS, each adult was asked, “Have you ever chewed tobacco, smoked a pipe, smoked self-rolled cigarettes, or smoked cigarettes/cigars?” and “Do you still have a smoking habit or have you totally stopped smoking?”. According to these two questions, all adults were divided into three mutually exclusive groups: never smoked, current smokers, and former smokers. For further analysis, the present study categorized current smokers into three subgroups (light, moderate, and heavy smokers) based on their average cigarette consumption (“In 1 day about how many cigarettes do you consume?”). Light smokers were current smokers who reported consuming from 1 to 10 cigarettes per day, moderate smokers were those who consumed from 11 to 19 cigarettes per day, and heavy smokers were those who consumed 20 cigarettes or more per day. Former smokers were categorized into three subgroups (short-term, moderate-term, and long-term quitters) based on the total number of years since the respondents had quit smoking (“At what age did you totally quit smoking?”): short-term quitters were former smokers who had quit smoking ≤ 1 year age, and moderate-term quitters and long-term quitters were former smokers who had quit smoking 2–5 years and ≥6 years age, respectively (42).

#### Covariates

The analysis also considered the following three categories of variables as covariates to explain depressive symptoms: (1) current health-related factors, including self-rated health, functional limitations, and chronic conditions; (2) several demographic characteristics that may also affect depressive symptoms, such as sex, age, educational attainment, marital status, and rural residency; and (3) in addition to smoking, another health behavior factor included in the analysis was drinking. The definitions of the variables are provided in Table 2.

**Table 2:** Definitions of the variables used in the empirical models.

**Variable**	**Description**
**Depressive symptoms**	1 if the individual self-rated CESD-10 score was equal to or greater than 10; 0 otherwise
**Smoking status**
Never smoked	1 if the individual who has never smoked; 0 otherwise
Short-term quitters	1 if the individual is former smoker who had quitted smoking ≤ 1 years; 0 otherwise
Moderate-term quitters	1 if the individual is former smoker who had quitted smoking 2–5 years; 0 otherwise
Long-term quitters	1 if the individual is former smoker who had quitted smoking ≥6 years; 0 otherwise
Light smokers	1 if the individual is current smoker who reported consuming from 1 to 10 cigarettes per day; 0 otherwise
Moderate smokers	1 if the individual is current smoker who reported consuming from 11 to 19 cigarettes per day; 0 otherwise
Heavy smokers	1 if the individual is current smoker who reported consuming 20 cigarettes or more per day; 0 otherwise
Sex	1 if the individual was male; 0 for females
Age	Continuous variable, actual age in years
**Educational attainment**
Illiteracy	1 if the individual was illiterate; 0 if otherwise
Elementary school	1 if the individual attended elementary school; 0 if otherwise
Middle school	1 if the individual graduated from middle school; 0 if otherwise
High school	1 if the individual graduated from high school; 0 if otherwise
Above 3-year of college	1 if the individual graduated from an above 3-year college; 0 if otherwise
**Marital status**
Never married	1 if the individual was never married; 0 if otherwise
Married	1 if the individual was married; 0 if otherwise
Separated or divorced	1 if the individual was separated or divorced; 0 if otherwise
Widowed	1 if the individual was widowed; 0 if otherwise
Rural residency	1 if the individual was rural resident; 0 if otherwise
**Self-rated health**
Poor	1 if the individual reported their health status to be poor or very poor; 0 if otherwise
Fair	1 if the individual reported their health status to be fair; 0 if otherwise
Good	1 if the individual reported their health status to be good or very good; 0 if otherwise
**Functionally limitations**
None	1 if the individual could eat, toilet, dress, bathe/shower, get in/out of bed, and walk without difficulty; 0 if otherwise
Mild	1 if the individual had one or two difficulties in eating, toileting, dressing, bathing/showering, getting in/out of bed, or walking; 0 if otherwise
Moderate	1 if the individual had three or four difficulties in eating, toileting, dressing, bathing/showering, getting in/out of bed, or walking; 0 if otherwise
Severe	1 if the individual had five to six difficulties in eating, toileting, dressing, bathing/showering, getting in/out of bed, or walking; 0 if otherwise
Chronic conditions	The number of chronic conditions diagnosed by a doctor.
Drinking	1 if the individual drank at least once a month; 0 if otherwise

### Multiple imputation of missing values

During the CHARLS investigation, the respondents were not required to answer any question in the cognition and depression section that they do not want to answer, and the interviewers went on to the next question. Moreover, the CESD-10 must be answered by the respondents themselves and cannot be answered by other family members. As a result, the proportion of missing values for the CESD-10 items was approximately 10% (see Table 1). In addition, the respondents who smoked filtered or unfiltered cigarettes answered the question about average cigarette consumption. The respondents who smoked a pipe, self-rolled cigarettes, cigars, or water cigarettes skipped the average cigarette consumption question. Therefore, missing values accounted for approximately 12% of the values for the variable of smoking status.

Testing on whether the given data set is MCAR or MAR was performed. The regular Little's MCAR test gives a distance of 22,619.85 with the degree of freedom = 10,099 and *p*-value < 0.001. The test suggests that the missing data of the measured variables of interest are not MCAR under significance level 0.05. Then we used a logistic model to identify other variables predicting missing responses to the CESD-10 items and smoking status (data not shown). The logistic model predicted that the missing data of the CESD-10 items and smoking status were related to the respondents' age. Therefore, the test provides evidence that the data is MAR. The present study adopted the MAR assumption and employed a multiple imputation technique to deal with missing values.

Multiple imputation is a simulation-based statistical technique that allows researchers to increase the availability of data points, thus reducing biases associated with the deletion of observations due to missing values (43). Multiple imputation has three elemental phases: imputation, analysis, and pooling. In the imputation phase, *m* copies of the dataset are created, with the missing values replaced by imputed values using an appropriate model. Rubin suggested that *m* = 5 should be sufficient to obtain valid inference (44). Therefore, 5 copies were created in this study to reduce the sampling error due to imputations.

Two common imputation approaches, multiple imputation with the multivariate normal model (MVN) and multiple imputation by chained equations (MICE), are widely available in statistical software. MVN assumes a joint multivariate normal distribution of all variables and uses multivariate normal data augmentation to impute missing values of imputation variables. MVN has a theoretical justification and appears to perform well compared to the MICE (45). However, most epidemiologists work with datasets that include non-continuous variables, which cannot be modeled by MVN (46). On the other hand, MICE is a more flexible approach that does not rely on rigorous theoretical justification to impute missing data for multiple variables based on a set of univariate imputation models (47). Therefore, the imputation process was carried out based on MICE. The present study selected conditional models based on the type of variables. MICE allows the use of logistic and Poisson regression models to impute binary variables, such as rural residency, and count variables, such as chronic conditions. Moreover, ordered logistic and multinomial logistic regression models can impute ordered categorical variables such as the CESD-10 items, educational attainment, and self-rated health, and unordered categorical variables, such as smoking status. Multiple imputation should include variables associated with the probability of missing values (48). The variables listed in Table 2 were used in the imputation models.

In the analysis phase, each of the 5 completed datasets was analyzed using a desired statistical method. The results obtained from 5 the completed datasets were combined into a single multiple-imputation result in the pooling phase. The single parameter estimate is calculated as the mean of the *m* (= 5) parameter estimates of $\hat{Q}$:


$$\bar{Q}=m^{-1}\overset{m}{\underset{i=1}{\sum }}{\hat{Q}}_{i}\;$$

The estimated variance of this MI estimate is calculated based on Rubin's rules as expressed below:


$$T=\bar{U}+\left(1+\frac{1}{m}\right)B\;$$

where $\bar{U}=m^{-1}\overset{m}{\underset{i=1}{\sum }}U_{i}$ is the estimated within imputation variance and *U*_*i*_ is the estimated variance from imputed data. $B={\left(m-1\right)}^{-1}\overset{m}{\underset{i=1}{\sum }}{\left({\hat{Q}}_{i}-\bar{Q}\right)}^{2}$ is the between imputation variance (37).

### Statistical analysis

Depressive symptoms may be both an antecedent and a consequence of tobacco smoking (10). Panel data, also referred to as longitudinal data in epidemiology, are a dataset in which observations of multiple subjects are collected over time. The benefit of panel data is that it is possible to control for the unknown or unmeasured determinants of depressive symptoms that are constant over time (49). Based on a four-wave unbalanced panel dataset, the current study estimated the effect of tobacco smoking on depressive symptoms and used a logistic regression model. The logistic regression model built a latent regression and was defined as follows:


$$y_{it}^{*}=x_{it}^{{}^{\prime }}\alpha +S_{it}^{{}^{\prime }}\beta +\varepsilon_{it},\;i=1,\ldots ,n,\;t=1,\ldots ,T_{i}\;$$

$y_{it}^{*}$ is an unobserved latent variable linked to the observed binary response variable (with or without depressive symptoms). $x_{it}^{{}^{\prime }}$ is the vector of the demographic characteristics and health status of an individual. The vector $S_{it}^{{}^{\prime }}$ represents smoking status including heavy smokers, moderate smokers, light smokers, short-term quitters, moderate-term quitters, and long-term quitters. α and β are the estimated coefficients. μ_*i*_ is the unobserved and individual-specific heterogeneity, and ε_*it*_ is a time-varying error term.

A logistic regression model was performed to analyze the impact of tobacco smoking on depressive symptoms among middle-aged and older adults in China. The first step in the analysis, pooled logistic regression, was a starting point. After that, this study treated the data as a panel structure and made a choice between the fixed effects and random effects logistic model. In this study, a possible unobserved variable was attitudes toward smoking, which was correlated with the time-varying explanatory variables (tobacco smoking) in the model. With such correlated heterogeneity, a fixed effects logistic model should be preferred over a random effects logistic model; however, when estimating a fixed effects logistic model, many pieces of information are lost. A random effects logistic model was also presented in this study (49, 50). The results are presented as odds ratios (ORs) along with 95% confidence intervals (CIs). All statistical analyses were conducted employing the Stata 15 statistical software package.

## Results

A descriptive summary of all variables over time is displayed in Table 3. The total sample size was 74,381 respondents, with 51.76% of the respondents being female and 32.61% aged 65 years or older. In addition, 77.88% of the respondents reported living in rural areas, and approximately 34% of the respondents completed at least middle school. The proportions of respondents with depressive symptoms were 37.55% in Wave 1, 32.26% in Wave 2, 33.68% in Wave 3, and 38.50% in Wave 4, implying that the proportions of respondents with depressive symptoms first decreased and then increased. Approximately one in four respondents has been current smokers over the years, and the proportions of light, moderate, and heavy smokers were approximately 9%, 2%, and 14%, respectively. Moreover, ~10% of the respondents were former smokers from 2011 to 2018. The proportions of short-term, moderate-term, and long-term quitters were approximately 2%, 2%, and 6%, respectively.

**Table 3:** Description of variables in four waves (percentage).

**Variable**	**Wave1 (2011)**	**Wave2 (2013)**	**Wave3 (2015)**	**Wave4 (2018)**	**All waves**
	***N* = 17,108**	***N* = 17,940**	***N* = 19,832**	***N* = 19,501**	***N* = 74,381**
**Depressive symptoms**
Yes	37.55	32.26	33.68	38.50	35.49
No	62.45	67.74	66.32	61.50	64.51
**Smoking status**
Never smoked	64.20	67.50	60.50	60.19	62.96
Short-term quitters	2.17	2.10	1.46	3.33	2.27
Moderate-term quitters	2.51	1.55	1.45	4.30	2.46
Long-term quitters	5.23	5.63	5.90	8.12	6.26
Light smokers	8.65	8.95	11.17	8.34	9.31
Moderate smokers	1.57	1.61	2.46	1.88	1.90
Heavy smokers	15.67	12.66	17.06	13.84	14.84
**Sex**
Male	48.68	48.31	48.47	47.56	48.24
Female	51.32	51.69	51.53	52.44	51.76
**Age**
45–64 years	72.48	69.37	67.49	60.99	67.39
65–84 years	26.34	29.16	30.96	36.89	31.02
≥85 years	1.18	1.47	1.55	2.12	1.59
**Educational attainment**
Illiterate	27.42	26.68	24.88	22.78	25.35
Elementary school	39.16	39.43	40.44	42.78	40.51
Middle school	20.67	20.85	21.48	21.80	21.23
High school	10.31	10.64	10.49	10.51	10.49
Above 3-year college	2.44	2.40	2.71	2.13	2.42
**Marital status**
Never married	0.89	0.85	0.78	0.61	0.78
Married	87.08	86.78	86.40	85.03	86.29
Separated or divorced	1.32	1.20	1.22	1.58	1.33
Widowed	10.71	11.17	11.60	12.78	11.60
**Rural residency**
Yes	77.48	76.57	78.02	79.28	77.88
No	22.52	23.43	21.98	20.72	22.12
**Self-rated health**
Poor	29.71	27.21	25.73	26.60	27.23
Fair	46.54	49.11	50.30	48.67	48.72
Good	23.75	23.68	23.97	24.73	24.05
**Functional limitations**
None	83.38	82.48	80.43	81.45	81.87
Mild	11.38	12.74	14.00	12.52	12.71
Moderate	3.27	3.24	3.67	3.76	3.50
Severe	1.97	1.54	1.90	2.27	1.92
**Chronic conditions**
Yes	67.26	66.96	61.45	79.35	68.81
No	32.74	33.04	38.55	20.65	31.19
**Drinking**
Yes	33.26	34.61	35.48	33.76	34.31
No	66.74	65.39	64.52	66.24	65.69

The results of the logistic regression analysis are shown in Table 4 as ORs. Column III of Table 4 presents the effect of tobacco smoking on depressive symptoms using the fixed effects logistic model. The results revealed that smoking status was associated with depressive symptoms. Heavy smokers had 20% higher odds of suffering from depressive symptoms than those who never smoked (OR = 1.20; 95% CI: 1.05, 1.37). Compared to those who never smoked, for short-term and moderate-term quitters, the odds of suffering from depressive symptoms increased 30% and 22%, respectively (OR = 1.30; 95% CI: 1.04, 1.63, OR = 1.22, 95% CI: 1.02, 1.47). In particular, long-term quitters had an increased likelihood of suffering from depressive symptoms than those who never smoked, but the difference was not statistically significant. The magnitudes of the ORs for the variables short-term quitters, moderate-term quitters, and long-term quitters decreased in absolute terms with increasing time-gaps since quitting. Therefore, among former smokers, the probability of suffering from depressive symptoms decreased with increasing duration of since quitting smoking.

**Table 4:** Logistic regression analysis of depressive symptoms.

	**Scenario 1**			**Scenario 2**
	**Pooled logistic**	**Random effects logistic**	**Fixed effects logistic**	**Fixed effects logistic**
	**(I) ORs (95% CI)**	**(II) ORs (95% CI)**	**III) ORs (95% CI)**	**(III) ORs (95% CI)**
**Smoking status**
Never smoked (ref.)	1.00	1.00	1.00	1.00
Short-term quitters	1.12 (0.97–1.28)	**1.19 (1.01–1.40)**	**1.30 (1.04–1.63)**	0.84 (0.64–1.09)
Moderate-term quitters	1.05 (0.93–1.19)	1.11 (0.95–1.29)	**1.22 (1.02–1.47)**	0.85 (0.66–1.09)
Long-term quitters	0.93 (0.82–1.05)	0.95 (0.82–1.10)	1.05 (0.88–1.26)	**0.82 (0.68–1.00)**
Light smokers	**1.14 (1.04–1.26)**	**1.17 (1.04–1.30)**	1.10 (0.96–1.27)	**0.66 (0.52–0.83)**
Moderate smokers	1.03 (0.88–1.21)	1.06 (0.89–1.27)	1.04 (0.83–1.29)	**0.65 (0.47–0.89)**
Heavy smokers	**1.14 (1.06–1.23)**	**1.20 (1.09–1.31)**	**1.20 (1.05–1.37)**	**0.72 (0.56–0.92)**
**Sex**
Male	**0.61 (0.57–0.66)**	**0.54 (0.49–0.59)**	0.88 (0.54–1.43)	1.10 (0.66–1.82)
Female (ref.)	1.00	1.00	1.00	1.00
**Age**	**0.99 (0.98–0.99)**	**0.99 (0.98–0.99)**	**1.01 (1.00–1.02)**	**1.04 (1.03–1.05)**
**Educational attainment**
Illiteracy (ref.)	1.00	1.00	1.00	1.00
Elementary school	**0.93 (0.88–0.98)**	**0.92 (0.86–0.98)**	1.11 (0.96–1.28)	1.01 (0.86–1.18)
Middle school	**0.70 (0.66–0.75)**	**0.65 (0.60–0.71)**	0.98 (0.78–1.25)	0.94 (0.74–1.18)
High school	**0.59 (0.54–0.65)**	**0.51 (0.46–0.57)**	0.82 (0.59–1.13)	0.85 (0.59–1.23)
Above 3-year college	**0.53 (0.44–0.64)**	**0.45 (0.36–0.55)**	0.89 (0.50–1.60)	0.93 (0.51–1.66)
**Marital status**
Never married (ref.)	1.00	1.00	1.00	1.00
Married	**0.64 (0.51–0.80)**	**0.57 (0.43–0.76)**	1.17 (0.50–2.78)	0.91 (0.37–2.24)
Separated or divorced	1.23 (0.94–1.61)	1.26 (0.90–1.76)	1.90 (0.78–4.64)	1.40 (0.55–3.54)
Widowed	0.92 (0.73–1.16)	0.91 (0.68–1.21)	1.96 (0.84–4.54)	1.52 (0.63–3.71)
**Rural residency**
Yes	**1.52 (1.43–1.61)**	**1.67 (1.55–1.79)**	1.08 (0.88–1.32)	1.01 (0.81–1.25)
No (ref.)	1.00	1.00	1.00	1.00
**Self-rated health**
Poor (ref.)	1.00	1.00	1.00	1.00
Fair	**0.42 (0.40–0.44)**	**0.37 (0.35–0.39)**	**0.52 (0.48–0.55)**	**0.58 (0.54–0.62)**
Good	**0.21 (0.20–0.22)**	**0.18 (0.16–0.19)**	**0.35 (0.32–0.38)**	**0.44 (0.40–0.48)**
**Functional limitations**
None (ref.)	1.00	1.00	1.00	1.00
Mild	**2.07 (1.96–2.18)**	**2.24 (2.10–2.39)**	**1.61 (1.49–1.74)**	**1.43 (1.31–1.56)**
Moderate	**3.63 (3.16–4.18)**	**4.49 (3.77–5.33)**	**2.91 (2.38–3.55)**	**2.74 (2.29–3.30)**
Severe	**4.98 (4.14–5.99)**	**7.28 (5.75–9.22)**	**4.97 (3.54–6.99)**	**5.97 (4.25–8.38)**
**Chronic conditions**
Yes	**1.15 (1.14–1.17)**	**1.20 (1.18–1.22)**	**1.09 (1.06–1.13)**	**1.07 (1.03–1.10)**
No (ref.)	1.00	1.00	1.00	1.00
**Drinking**
Yes	1.01 (0.97–1.06)	1.01 (0.95–1.06)	1.02 (0.94–1.11)	0.93 (0.85–1.02)
No (ref.)	1.00	1.00	1.00	1.00
Observations	74,381	74,381	35,466	27,456

Scenario 1: Apply multiple imputation to account for missing responses [seed for random number generator (54,321)]. Scenario 2: Estimate without accounting for missing responses (listwise deletion/complete case analysis). Bold indicates statistical significance, p < 0.05.

Irrespective of the estimation method in Scenario 1, heavy smokers showed an increased probability of suffering from depressive symptoms compared to those who had never smoked. Short-term quitters showed an increased probability of suffering from depressive symptoms compared to those who had never smoked (see Columns I-III of Table 4). The missing values were not imputed in Scenario 2, the results indicated that former smokers showed a decreased probability of suffering from depressive symptoms compared to those who had never smoked.

The complete case and multiple imputation analyses are shown in Table 5. The coefficient of variation (CV) of the OR offers its normalized measure of dispersion. Compared to complete case analysis, for smoking status variables, it is clearly reduced after multiple imputation. The variation rate (VR) assesses the relative variation of the OR obtained from complete case and multiple imputation analyses. For smoking status variables, the VR varied from 28.05% for long-term quitters to 66.67% for light and heavy smokers.

**Table 5:** Fixed effects logistic regression analysis for the complete case and multiple imputation.

	**Complete case**				**Multiple imputation**				
	**aORs**	**95% CI**	**SE**	**CV(%)**	**aORs**	**95% CI**	**SE**	**CV(%)**	**VR_MI_(%)**
**Smoking status**
Never smoked (ref.)	1.00				1.00				
Short-term quitters	0.84	0.64–1.09	0.11	13.10	**1.30**	**1.04–1.63**	0.14	10.77	54.76
Moderate-term quitters	0.85	0.66–1.09	0.11	12.94	**1.22**	**1.02–1.47**	0.11	9.02	43.53
Long-term quitters	**0.82**	**0.68–1.00**	0.08	9.76	1.05	0.88–1.26	0.09	8.57	28.05
Light smokers	**0.66**	**0.52–0.83**	0.08	12.12	1.10	0.96–1.27	0.08	7.27	66.67
Moderate smokers	**0.65**	**0.47–0.89**	0.10	15.38	1.04	0.83–1.29	0.11	10.58	60.00
Heavy smokers	**0.72**	**0.56–0.92**	0.09	12.50	**1.20**	**1.05–1.37**	0.08	6.67	66.67

aORs, odds ratios adjusted on sex, age, educational attainment, marital status, rural residency, self-rated health, functional limitations, chronic conditions, and drinking. CV, the coefficient of variation. VR_MI_, the variation rate for complete case and multiple imputation analyses. Bold indicates statistical significance, p < 0.05.

The smoking prevalence in China varies widely between men and women. Therefore, the current study conducted a sub-group analysis for men and women, respectively (see Table 6). The results of the fixed effects logistic model revealed that heavy male smokers, short-term and moderate-term male quitters had higher odds of suffering from depressive symptoms than those who never smoked (OR = 1.22; 95% CI: 1.05, 1.41, OR = 1.31; 95% CI: 1.05, 1.64, OR = 1.25; 95% CI: 1.02, 1.54). However, associations between smoking status and depressive symptoms were not significant for women.

**Table 6:** Fixed effects logistic regression analysis of depressive symptoms stratified by male-female.

	**Male**	**Female**
	**aORs (95% CI)**	**aORs (95% CI)**
**Smoking status**
Never smoked (ref.)	1.00	1.00
Short-term quitters	**1.31 (1.05–1.64)**	1.45 (0.73–2.89)
Moderate-term quitters	**1.25 (1.02–1.54)**	1.17 (0.59–2.29)
Long-term quitters	1.06 (0.88–1.29)	1.15 (0.77–1.71)
Light smokers	1.13 (0.97–1.31)	1.07 (0.75–1.52)
Moderate smokers	1.05 (0.84–1.32)	0.98 (0.49–1.96)
Heavy smokers	**1.22 (1.05–1.41)**	1.07 (0.69–1.65)

aORs, odds ratios adjusted on sex, age, educational attainment, marital status, rural residency, self-rated health, functional limitations, chronic conditions, and drinking. Bold indicates statistical significance, p < 0.05.

## Discussion

To the best of our knowledge, this is the first research study that examined the effect of tobacco smoking on depressive symptoms among individuals aged 45 years or older in China using a nationally representative survey with a multiple imputation technique. The results indicated that among Chinese middle-aged and older adults, heavy smokers and short-term and moderate-term quitters have increased odds of suffering from depressive symptoms than those who never smoked after controlling for other relevant variables. Our findings are consistent with the findings for European middle-aged and older adults (16) and American middle-aged adults (17). Therefore, local communities and primary care facilities should consider promoting health education programs for middle-aged and older smokers and improving their understanding of the hazards of tobacco smoking.

This type of analysis, however, does not identify pathways between tobacco smoking and depressive symptoms. One possible explanation is that smoking or chewing tobacco releases nicotine affecting an individual's neurocircuitry, which increases their susceptibility to depression (13, 15). Another possible explanation is from a self-medication model, suggesting that smokers use nicotine to alleviate depressed mood (9, 18, 51).

This study found that short-term and moderate-term quitters show increased odds of suffering from depressive symptoms than those who never smoked regardless of the time elapsed since quitting, but the likelihood of having depressive symptoms declined with increasing time gaps since stopping smoking. Although the causality between smoking cessation and depression cannot be established without a longitudinal follow-up design, the decline in the prevalence of depressive symptoms after 1 year of quitting implies that smoking cessation and depressive symptoms are related. These results are comparable to other findings in the literature (52–54). In this study, former smokers reported that the probability of having depressive symptoms decreased with a longer duration since quitting, and hence, the earlier a smoker stops smoking, the greater the impact of smoking cessation on not being depressed. In other words, smoking cessation is an effective way to reduce middle-aged and older adults' risk of depression. Therefore, the government should consider allocating more resources to smoking cessation programs to help adults and adolescent smokers quit as early as possible and/or to remain non-smokers. It is also essential to tailor smoking cessation programs for middle-aged and older adults and help them quit smoking and prevent relapse.

The sub-group analysis for men and women found that associations between smoking status and depressive symptoms were significant for men but not women. Therefore, the association between depressive symptoms and smoking among Chinese middle-aged and older adults is not straightforward and may vary according to gender. According to the multiple imputed datasets, we found that the proportion of current smoking in men (54.17%) was significantly higher than in women (5.39%). However, the proportion of women with depressive symptoms (42.26%) was significantly higher than in men (28.23%). The results revealed that men are more likely to smoke and women are more likely to suffer from depressive symptoms. Given the special situation, further study will be needed to employ different types of research methods investigating gender differences in the association between depressive symptoms and smoking.

Extensive health-related surveys, such as the CHARLS, provide numerous data regarding health-related behaviors and health outcomes. However, almost every analysis faces the annoying problem of missing data. When comparing the results from multiple imputation and listwise deletion (Table 4). multiple imputation recovers a fully observed sample size. More importantly, multiple imputation restores the natural variability of the missing values. Recovering information and restoring variability may reduce bias or increase precision, which results in a valid statistical inference from multiple imputation (35, 55). It will be essential to employ multiple imputation in future analyses, when survey items on smoking status and depressive symptoms have a large number of missing values. However, researchers should be aware of hazards in multiple imputation analyses. First, multiple imputation is valid under the MAR assumption and gives biased results for MNAR mechanisms. Unfortunately, the distinction between MAR and MNAR is based on a non-testable assumption. Second, the misspecification of the imputation model gives biased results unless enough variables predictive of missing data are included in the imputation model (56–58).

### Limitations

Although the current study employs a national survey with multiple imputation to analyze the effect of smoking status on depressive symptoms in Chinese middle-aged and older adults, several limitations should be emphasized. First, the results provide no evidence about the causal direction between smoking and depressive symptoms. The authors can only conclude that findings suggest the co-occurrence of smoking and depressive symptoms among Chinese middle-aged and older adults. Second, since the CESD-10 is a self-reported screening instrument for symptoms of depression and not a diagnostic tool, our analysis may have resulted in either an underestimation or overestimation of the association between tobacco smoking and depressive symptoms. Third, the data were obtained *via* surveys, and thus the limitations of all self-reported data exist, such as recall bias and the unreliability of responses when respondents are under pressure. Last, when the respondents' unchanging depressive symptoms across all four waves did not contribute to the likelihood, the results of the fixed effects model could be less precise and have larger standard errors.

## Conclusion

The purpose of this study was to empirically ascertain the effect of tobacco smoking on depressive symptoms among individuals aged 45 years or older in China using multiple imputed datasets. The empirical findings suggest that among Chinese middle-aged and older adults, heavy smokers and short-term and moderate-term quitters have increased odds of suffering from depressive symptoms than those who never smoked. Moreover, former smokers reported that the probability of having depressive symptoms decreased with a longer duration since quitting. Nevertheless, the association between depressive symptoms and smoking among Chinese middle-aged and older adults is not straightforward and may vary according to gender. These results may have important implications that support the government in allocating more resources to smoking cessation programs to help middle-aged and older smokers, particularly in men.

## Data availability statement

Publicly available datasets were analyzed in this study. This data can be found here: https://opendata.pku.edu.cn/dataverse/CHARLS.

## Author contributions

XD and CL collaboratively designed the study, developed the methodology, and interpreted the results. CL and XD led the data analysis and wrote the manuscript. RW, LK, and LZ made important contributions to the revision of the manuscript. All authors read and approved the final manuscript.

## Funding

This research was funded by the Inner Mongolia Autonomous Region Natural Science Fund (2021BS07006). The funding bodies played no role in the design of the study, the collection, analysis, and interpretation of the data or the in writing of the manuscript.

## Conflict of interest

The authors declare that the research was conducted in the absence of any commercial or financial relationships that could be construed as a potential conflict of interest.

## Publisher's note

All claims expressed in this article are solely those of the authors and do not necessarily represent those of their affiliated organizations, or those of the publisher, the editors and the reviewers. Any product that may be evaluated in this article, or claim that may be made by its manufacturer, is not guaranteed or endorsed by the publisher.

## Abbreviations

CESD-10: 10-item Center for Epidemiologic Studies Depression Scale
MCAR: missing completely at random
MAR: missing at random
MNAR: missing not at random
CHARLS: China Health and Retirement Longitudinal Study
CESD-D: 20-item Center for Epidemiologic Studies Depression Scale
MICE: multiple imputation by chained equations
OR: odds ratio
CI: confidence interval.

## References

[1] World Health Organization. Tobacco. (2021). Available online at: https://www.who.int/news-room/fact-sheets/detail/tobacco (accessed January 12, 2022).

[2] World Health Organization. Global Adult Tobacco Survey (GATS). Fact sheet China 2018. (2019). Available online at: https://www.who.int/docs/default-source/wpro—documents/countries/china/2018-gats-china-factsheet-cn-en.pdf (accessed January 10, 2022).

[3] Liu Q He H Yang J Feng X Zhao F Lyu J. Changes in the global burden of depression from 1990 to 2017: Findings from the Global Burden of Disease study. J Psychiatr Res. (2020) 126:134–40. 10.1016/j.jpsychires.2019.08.002 31439359

[4] Blazer DG. Depression in late life: review and commentary. J Gerontol A Biol Sci Med Sci. (2003) 58:249–65. 10.1093/gerona/58.3.M249 12634292

[5] Tang T Jiang J Tang X. Prevalence of depressive symptoms among older adults in mainland China: a systematic review and meta-analysis. J Affect Disord. (2021) 293:379–90. 10.1016/j.jad.2021.06.050 34246000

[6] Stanton R To QG Khalesi S Williams SL Alley SJ Thwaite TL. Depression, anxiety and stress during COVID-19: associations with changes in physical activity, sleep, tobacco and alcohol use in Australian adults. Int J Environ Res Public Health. (2020) 17:4065. 10.3390/ijerph17114065 32517294 PMC7312903

[7] Tzu-Hsuan Chen D. The psychosocial impact of the COVID-19 pandemic on changes in smoking behavior: Evidence from a nationwide survey in the UK. Tob Prev Cessat. (2020) 6:59. 10.18332/tpc/126976 33163705 PMC7643580

[8] Audrain-McGovern J Rodriguez D Kassel JD. Adolescent smoking and depression: evidence for self-medication and peer smoking mediation. Addiction. (2009) 104:1743–56. 10.1111/j.1360-0443.2009.02617.x 19549056 PMC2891382

[9] Chaiton MO Cohen JE O'Loughlin J Rehm J. A systematic review of longitudinal studies on the association between depression and smoking in adolescents. BMC Public Health. (2009) 9:356. 10.1186/1471-2458-9-356 19772635 PMC2758872

[10] Liew HP Gardner S. The interrelationship between smoking and depression in Indonesia. Health Policy Technol. (2016) 5:26–31. 10.1016/j.hlpt.2015.10.003

[11] An R. Xiang X. Smoking, heavy drinking, and depression among US middle-aged and older adults. Prev Med. (2015) 81:295–302. 10.1016/j.ypmed.2015.09.026 26436684

[12] Wootton RE Richmond RC Stuijfzand BG Lawn RB Sallis HM. Taylor GMJ. Evidence for causal effects of lifetime smoking on risk for depression and schizophrenia: a Mendelian randomisation study. Psychol Med. (2020) 50:2435–43. 10.1017/S0033291719002678 31689377 PMC7610182

[13] Boden JM Fergusson DM Horwood LJ. Cigarette smoking and depression: tests of causal linkages using a longitudinal birth cohort. Br J Psychiatry. (2010) 196:440–6. 10.1192/bjp.bp.109.065912 20513853

[14] Kang E Lee J. A longitudinal study on the causal association between smoking and depression. J Prev Med Public Health. (2010) 43:193–204. 10.3961/jpmph.2010.43.3.193 20534959

[15] Wiesbeck GA Kuhl HC Yaldizli O Wurst FM WHO/ISBRA WHO/ISBRA Study Group on Biological State and Trait Markers of Alcohol Use and Dependence. Tobacco smoking and depression–results from the WHO/ISBRA study. Neuropsychobiology. (2008) 57:26–31. 10.1159/000123119 18424908

[16] Werneck AO Peralta M Tesler R Marques A. Cross-sectional and prospective associations of lifestyle risk behaviors clustering with elevated depressive symptoms among middle-aged and older adults. Maturitas. (2022) 155:8–13. 10.1016/j.maturitas.2021.09.010 34876251

[17] Johnson EO Breslau N. Is the association of smoking and depression a recent phenomenon? Nicotine Tob Res. (2006) 8:257–62. 10.1080/14622200600576644 16766418

[18] Lam TH Li ZB Ho SY Chan WM Ho KS Li MP. Smoking and depressive symptoms in Chinese elderly in Hong Kong. Acta Psychiatr Scand. (2004) 110:195–200. 10.1111/j.1600-0447.2004.00342.x 15283739

[19] Magee W Clarke P. The effect of smoking on depressive symptoms. Addict Behav. (2021) 112:106641. 10.1016/j.addbeh.2020.106641 33010527

[20] Cheng HG Chen S McBride O Phillips MR. Prospective relationship of depressive symptoms, drinking, and tobacco smoking among middle-aged and elderly community-dwelling adults: Results from the China Health and Retirement Longitudinal Study (CHARLS). J Affect Disord. (2016) 195:136–43. 10.1016/j.jad.2016.02.023 26895091

[21] Holahan CK Holahan CJ Powers DA Hayes RB Marti CN Ockene JK. Depressive symptoms and smoking in middle-aged and older women. Nicotine Tob Res. (2011) 13:722–31. 10.1093/ntr/ntr066 21504881 PMC3150691

[22] Fergusson DM Goodwin RD Horwood LJ. Major depression and cigarette smoking: results of a 21-year longitudinal study. Psychol Med. (2003) 33:1357–67. 10.1017/S0033291703008596 14672244

[23] Weinberger AH Kashan RS Shpigel DM Esan H Taha F Lee CJ. Depression and cigarette smoking behavior: A critical review of population-based studies. Am J Drug Alcohol Abuse. (2017) 43:416–31. 10.3109/00952990.2016.1171327 27286288

[24] Stubbs B Vancampfort D Firth J Solmi M Siddiqi N Smith L. Association between depression and smoking: a global perspective from 48 low- and middle-income countries. J Psychiatr Res. (2018) 103:142–9. 10.1016/j.jpsychires.2018.05.018 29852421

[25] Acartürk CZ Nierkens V Agyemang C Stronks K. Depressive symptoms and smoking among young Turkish and Moroccan ethnic minority groups in The Netherlands: a cross-sectional study. Subst Abuse Treat Prev Policy. (2011) 6:5. 10.1186/1747-597X-6-5 21414199 PMC3064616

[26] Schuler MS Vasilenko SA Lanza ST. Age-varying associations between substance use behaviors and depressive symptoms during adolescence and young adulthood. Drug Alcohol Depend. (2015) 157:75–82. 10.1016/j.drugalcdep.2015.10.005 26483358 PMC4663168

[27] Cicchetti D Toth SL. The development of depression in children and adolescents. Am Psychol. (1998) 53:221–41. 10.1037/0003-066X.53.2.221 9491749

[28] Kenney BA Holahan CJ Holahan CK Brennan PL Schutte KK Moos RH. Depressive symptoms, drinking problems, and smoking cessation in older smokers. Addict Behav. (2009) 34:548–53. 10.1016/j.addbeh.2009.03.020 19372009 PMC2752429

[29] Malhi GS Mann JJ. Depression. Lancet. (2018) 392:2299–312. 10.1016/S0140-6736(18)31948-2 30396512

[30] Fan X Guo X Ren Z Li X He M Shi H. The prevalence of depressive symptoms and associated factors in middle-aged and elderly Chinese people. J Affect Disord. (2021) 293:222–8. 10.1016/j.jad.2021.06.044 34217959

[31] Liu Q Cai H Yang LH Xiang YB Yang G Li H. Depressive symptoms and their association with social determinants and chronic diseases in middle-aged and elderly Chinese people. Sci Rep. (2018) 8:3841. 10.1038/s41598-018-22175-2 29497126 PMC5832867

[32] Wen Y Liu C Liao J Yin Y Wu D. Incidence and risk factors of depressive symptoms in 4 years of follow-up among mid-aged and elderly community-dwelling Chinese adults: findings from the China Health and Retirement Longitudinal Study. BMJ Open. (2019) 9:e029529. 10.1136/bmjopen-2019-029529 31501114 PMC6738713

[33] Shrive FM Stuart H Quan H Ghali WA. Dealing with missing data in a multi-question depression scale: a comparison of imputation methods. BMC Med Res Methodol. (2006) 6:57. 10.1186/1471-2288-6-57 17166270 PMC1716168

[34] Andresen EM Byers K Friary J Kosloski K Montgomery R. Performance of the 10-item center for epidemiologic studies depression scale for caregiving research. SAGE Open Med. (2013) 1:2050312113514576. 10.1177/2050312113514576 26770693 PMC4687763

[35] Kang H. The prevention and handling of the missing data. Korean J Anesthesiol. (2013) 64:402–6. 10.4097/kjae.2013.64.5.402 23741561 PMC3668100

[36] Rubin DB. Inference and missing data. Biometrika. (1976) 63:581–92. 10.1093/biomet/63.3.581

[37] Pedersen AB Mikkelsen EM Cronin-Fenton D Kristensen NR Pham TM Pedersen L. Missing data and multiple imputation in clinical epidemiological research. Clin Epidemiol. (2017) 9:157–66. 10.2147/CLEP.S129785 28352203 PMC5358992

[38] Zhao Y Hu Y Smith JP Strauss J Yang G. Cohort profile: the China Health and Retirement Longitudinal Study (CHARLS). Int J Epidemiol. (2014) 43:61–8. 10.1093/ije/dys203 23243115 PMC3937970

[39] Andresen EM Malmgren JA Carter WB Patrick DL. Screening for depression in well older adults: evaluation of a short form of the CES-D (Center for Epidemiologic Studies Depression Scale). Am J Prev Med. (1994) 10:77–84. 10.1016/S0749-3797(18)30622-6 8037935

[40] Cheng ST Chan AC. The center for epidemiologic studies depression scale in older chinese: thresholds for long and short forms. Int J Geriatr Psychiatry. (2005) 20:465–70. 10.1002/gps.1314 15852439

[41] Ossip-Klein D Rothenberg B Andresen E. Depression screening measures. In: Andresen E, Rothenberg B, Zimmer J, editors. Assessing the Health Status of Older Adults. New York, NY: Springer Publishing Co. (1997). p. 180–244.

[42] Canada Government. Tobacco Use Statistics. (2008). Available online at: https://www.canada.ca/en/health-canada/services/health-concerns/tobacco/research/tobacco-use-statistics/terminology.html (accessed December 20, 2021).

[43] Penn DA. Estimating missing values from the general social survey: An application of multiple imputation. Soc Sci Quart. (2007) 88:573–84. 10.1111/j.1540-6237.2007.00472.x

[44] Rubin DB. Multiple Imputation for Nonresponse in Surveys. New York, NY: John Wiley & Sons (1987).

[45] Lee KJ Carlin JB. Multiple imputation for missing data: fully conditional specification versus multivariate normal imputation. Am J Epidemiol. (2010) 171:624–32. 10.1093/aje/kwp425 20106935

[46] Kropko J Goodrich B Gelman A Hill J. Multiple imputation for continuous and categorical data: comparing joint multivariate normal and conditional approaches. Polit Anal. (2014) 22:497–519. 10.1093/pan/mpu007

[47] White IR Royston P Wood AM. Multiple imputation using chained equations: Issues and guidance for practice. Stat Med. (2011) 30:377–99. 10.1002/sim.4067 21225900

[48] Spratt M Carpenter J Sterne JA Carlin JB Heron J Henderson J. Strategies for multiple imputation in longitudinal studies. Am J Epidemiol. (2010) 172:478–87. 10.1093/aje/kwq137 20616200

[49] Andreß HJ Golsch K Schmidt AW. Applied Panel Data Analysis for Economic and Social Surveys. Berlin: Springer Science and Business Media (2013).

[50] Greene WH. Econometric Analysis. 7th ed. International edition New Jersey: Prentice Hall (2012).

[51] Fluharty M Taylor AE Grabski M Munafò MR. The association of cigarette smoking with depression and anxiety: a systematic review. Nicotine Tob Res. (2017) 19:3–13. 10.1093/ntr/ntw140 27199385 PMC5157710

[52] McClave AK Dube SR Strine TW Kroenke K Caraballo RS Mokdad AH. Associations between smoking cessation and anxiety and depression among US adults. Addict Behav. (2009) 34:491–7. 10.1016/j.addbeh.2009.01.005 19217720

[53] Piper ME Rodock M Cook JW Schlam TR Fiore MC Baker TB. Psychiatric diagnoses among quitters versus continuing smokers 3 years after their quit day. Drug Alcohol Depend. (2013) 128:148–54. 10.1016/j.drugalcdep.2012.08.023 22995766 PMC3591817

[54] Prochaska JJ Hall SM Tsoh JY Eisendrath S Rossi JS Redding CA. Treating tobacco dependence in clinically depressed smokers: effect of smoking cessation on mental health functioning. Am J Public Health. (2008) 98:446–8. 10.2105/AJPH.2006.101147 17600251 PMC2253568

[55] Lee KJ Carlin JB. Recovery of information from multiple imputation: a simulation study. Emerg Themes Epidemiol. (2012) 9:3. 10.1186/1742-7622-9-3 22695083 PMC3544721

[56] Hughes RA Heron J Sterne JAC Tilling K. Accounting for missing data in statistical analyses: multiple imputation is not always the answer. Int J Epidemiol. (2019) 48:1294–304. 10.1093/ije/dyz032 30879056 PMC6693809

[57] Sterne JA White IR Carlin JB Spratt M Royston P Kenward MG. Multiple imputation for missing data in epidemiological and clinical research: potential and pitfalls. BMJ. (2009) 338:b2393. 10.1136/bmj.b2393 19564179 PMC2714692

[58] Sinharay S Stern HS Russell D. The use of multiple imputation for the analysis of missing data. Psychol Methods. (2001) 6:317–29. 10.1037/1082-989X.6.4.317 11778675

